# Comparative analysis of Wnt expression identifies a highly conserved developmental transition in flatworms

**DOI:** 10.1186/s12915-016-0233-x

**Published:** 2016-03-04

**Authors:** Uriel Koziol, Francesca Jarero, Peter D. Olson, Klaus Brehm

**Affiliations:** Sección Bioquímica, Facultad de Ciencias, Universidad de la República, Montevideo, Uruguay; University of Würzburg, Institute for Hygiene and Microbiology, Würzburg, Germany; Department of Life Sciences, The Natural History Museum, London, UK

**Keywords:** Antero-posterior axis, Cestodes, FoxQ2, Metamorphosis, Myocyte, Phylotypic, Planarian, Platyhelminthes, SFRP, Six3/6, Wnt

## Abstract

**Background:**

Early developmental patterns of flatworms are extremely diverse and difficult to compare between distant groups. In parasitic flatworms, such as tapeworms, this is confounded by highly derived life cycles involving indirect development, and even the true orientation of the tapeworm antero-posterior (AP) axis has been a matter of controversy. In planarians, and metazoans generally, the AP axis is specified by the canonical Wnt pathway, and we hypothesized that it could also underpin axial formation during larval metamorphosis in tapeworms.

**Results:**

By comparative gene expression analysis of Wnt components and conserved AP markers in the tapeworms *Echinococcus multilocularis* and *Hymenolepis microstoma*, we found remarkable similarities between the early stages of larval metamorphosis in tapeworms and late embryonic and adult development in planarians. We demonstrate posterior expression of specific Wnt factors during larval metamorphosis and show that scolex formation is preceded by localized expression of Wnt inhibitors. In the highly derived larval form of *E. multilocularis*, which proliferates asexually within the mammalian host, we found ubiquitous expression of posterior Wnt factors combined with localized expression of Wnt inhibitors that correlates with the asexual budding of scoleces. As in planarians, muscle cells are shown to be a source of secreted Wnt ligands, providing an explanation for the retention of a muscle layer in the immotile *E. multilocularis* larva.

**Conclusions:**

The strong conservation of gene expression between larval metamorphosis in tapeworms and late embryonic development in planarians suggests, for the first time, a homologous developmental period across this diverse phylum. We postulate these to represent the phylotypic stages of these flatworm groups. Our results support the classical notion that the scolex is the true anterior end of tapeworms. Furthermore, the up-regulation of Wnt inhibitors during the specification of multiple anterior poles suggests a mechanism for the unique asexual reproduction of *E. multilocularis* larvae.

**Electronic supplementary material:**

The online version of this article (doi:10.1186/s12915-016-0233-x) contains supplementary material, which is available to authorized users.

## Background

Flatworms (Platyhelminthes) are a highly diverse and ubiquitous phylum of dorso-ventrally flattened animals that include a wide range of free-living and symbiotic forms. The majority of described species are obligate parasites belonging to the Neodermata, a monophyletic group that includes monogeneans, trematodes (flukes), and cestodes (tapeworms). The singular origin of neodermatan worms represents one of the most successful evolutionary transitions to parasitism seen in the animal kingdom [1–3].

The Neodermata have complex life cycles with one or more larval stages. Tapeworms (Cestoda) are particularly derived in their morphology and development, as they lack a gut and any trace of endoderm during embryogenesis [4]. The early embryonic development of tapeworms consists of asynchronous and asymmetric divisions, and there is no process resembling gastrulation [5–7]. The final product of embryogenesis is a highly reduced larval form, the oncosphere [7, 8]. This larva is passively taken up by an intermediate host, and is specialized for penetrating through the gut wall by means of six moving hooks and the secretions of penetration glands. The oncosphere metamorphoses into the next life stage, the metacestode, in a parenteral site of the first host. Most of the oncospheral cells are discarded, and the metacestode tissues are generated from a few stem cells that were set aside during embryogenesis [5, 8]. The metacestode consists of an anterior end, the scolex, which contains attachment organs, and a posterior undifferentiated body. Once the metacestode is ingested by the definitive host, it attaches to the wall of the gut, and develops into the adult form by continuously generating segments from the neck region behind the scolex. Within each segment, male and female reproductive systems develop, resulting in the generation of eggs by sexual reproduction.

**Fig. 1:**
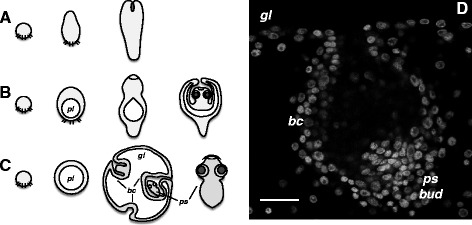
The oncosphere to metacestode metamorphosis in different tapeworms. A generalized summary of development is shown for different tapeworm groups. **a** Development in basal eucestodes, without the formation of an inner cavity. **b** Development in most cyclophyllidean tapeworms, with the formation of a cavity (primary lacuna, *pl*) during early development. The scolex invaginates or retracts into this cavity during later development. **c** Development in *Echinococcus multilocularis*. The tissues surrounding the primary lacuna expand into the thin germinal layer (*gl*), from which many brood capsules (*bc*) invaginate. The protoscoleces (*ps*) develop within the brood capsules, and are released when the metacestode is ingested by the definitive host. **d** Nuclear staining with 4’,6-diamidino-2-phenylindole (DAPI) of an *E. multilocularis* metacestode vesicle from *in vitro* culture, showing the continuity of the germinal layer (*gl*), brood capsule (*bc*) and protoscolex bud (*ps bud*). Bar: 20 μm

Because of the unique morphology and development of tapeworms, it has been impossible to make meaningful comparisons with other flatworms, let alone other animal phyla. Even the true polarity of the tapeworm antero-posterior (AP) axis has been a matter of controversy [9, 10]. Conventionally, the scolex is taken to be the anterior of the adult, based on its functional orientation and on the centralization of the nervous system within it. However, it has been postulated that this accumulation of nervous elements could simply be due to the requirements for innervation of the muscular attachment organs of the scolex. Furthermore, the relative position of the male and female gonads in each segment (male gonads closer to the scolex) would be opposite that found in free-living flatworms if the scolex is considered anterior. Perhaps the most controversial point involves the relative polarity of the oncosphere and the adult; during metamorphosis, the scolex is formed at the pole of the oncosphere opposite to that containing the hooks (the “functional anterior end” of the oncosphere, Fig. 1), and therefore it has been postulated that a reversal of polarity occurs during this metamorphosis [8, 9].

It has recently been shown (free-living flatworms) that canonical Wnt/β-catenin signaling is involved in the specification and maintenance of the AP axis in planarians during regeneration and during normal tissue turnover, similar to what happens during early development in most bilaterian animals [11–15]. Specific Wnt ligands are expressed in gradients from the posterior end, and signaling through this pathway is required to maintain and specify the posterior of adult planarians. Conversely, extracellular inhibitors of Wnts are specifically expressed in the anterior end, and inhibition of canonical Wnt signaling is necessary for anterior specification.

We speculated that, although early development is very divergent between tapeworms and planarians, conservation of a fundamental AP specification program should be shared across the phylum. Thus, the later stages of planarian development should show homologous gene expression patterns to the oncosphere-to-metacestode metamorphosis in tapeworms, as these are the stages where the main body plan is established, whereas earlier developmental stages are specialized for their specific ecological requirements [8, 16].

In this work, we elucidate the expression patterns of Wnt ligands and inhibitors as well as other highly conserved animal markers of AP polarity during metacestode development in the tapeworms *Echinococcus multilocularis* and *Hymenolepis microstoma*. Our results show a striking conservation of gene expression, leading us to propose that these stages represent the phylotypic period of flatworms, and that the scolex is the true anterior end of tapeworms. Furthermore, our results indicate that the unique development of the *E. multilocularis* metacestode is the result of a lack of AP polarity during early development, resulting in a completely posteriorized metacestode from which multiple foci of anterior development subsequently arise.

## Results

### A re-interpretation of *E. multilocularis* larval morphology and development

The life cycle outlined in the introduction is a generalization of the diversity found in the ‘true’ tapeworms, the Eucestoda (Fig. 1a). However, variations of this plan occur in particular groups. Many species of the order Cyclophyllidea form an internal cavity during early metacestode development, called the primary lacuna (Fig. 1b) [8, 17, 18]. The tissues surrounding this cavity form a cyst or bladder. The scolex retracts or invaginates into this cavity, and is protected by the surrounding tissue (Fig. 1b).

Among cyclophyllideans, metacestodes of *Echinococcus* spp. are the causative agents of dangerous zoonoses worldwide [19], and display unique development in their intermediate hosts (typically in the liver) (Fig. 1c) [17, 20]. Initially, only bladder tissue is generated from the oncosphere, forming a large vesicle that is filled with fluid, and contains only a thin layer of tissue in the periphery (the germinal layer) (Fig. 1c). From the germinal layer, secondary vesicles, called brood capsules, are formed towards the inner cavity. Within the brood capsules, nascent scoleces (protoscoleces) are formed by budding, resulting in massive asexual propagation (Fig. 1c). The protoscoleces are attached to the brood capsule by a thin stalk, and are released when the metacestode is ingested by the definitive host.

The evolutionary origin of the unique *E. multilocularis* metacestode remains unsolved. This is partly because the development of *E. multilocularis* has been historically regarded to be fundamentally different from that of other tapeworms, as the protoscoleces were considered to form within the central cavity, towards its interior (endogenous development), as opposed to forming on the external surface of the metacestode as in most other tapeworms (exogenous development) [8, 21–23]. However, we have recently shown, using confocal microscopy, that brood capsules and protoscoleces of *E. multilocularis* are actually formed from an invagination of the germinal layer of metacestode vesicles, and therefore just as in other tapeworms, the scolex is formed from the metacestode body wall towards the exterior [24] (Fig. 1c,d; see also [25, 26]). If one assumes that the scolex is the anterior end of tapeworms, then the *E. multilocularis* metacestode can be interpreted as showing differentiation along an AP axis, with many anterior ends (protoscoleces) followed along the AP axis by the brood capsules, and finally converging to one common posterior represented by the germinal layer of the vesicles. Therefore, we hypothesized that, during early development, the *E. multilocularis* metacestode is composed exclusively of posterior tissues and anterior development is suppressed. The remaining stages of metacestode differentiation are delayed, and only later do multiple foci of anterior development arise from the germinal layer. This is in contrast to most tapeworms, in which a single scolex forms very early during development, always in the region opposite to the hooks of the oncosphere [8].

### Expression of Wnt and SFRP genes during early metacestode development of *E. multilocularis*

**Fig. 2:**
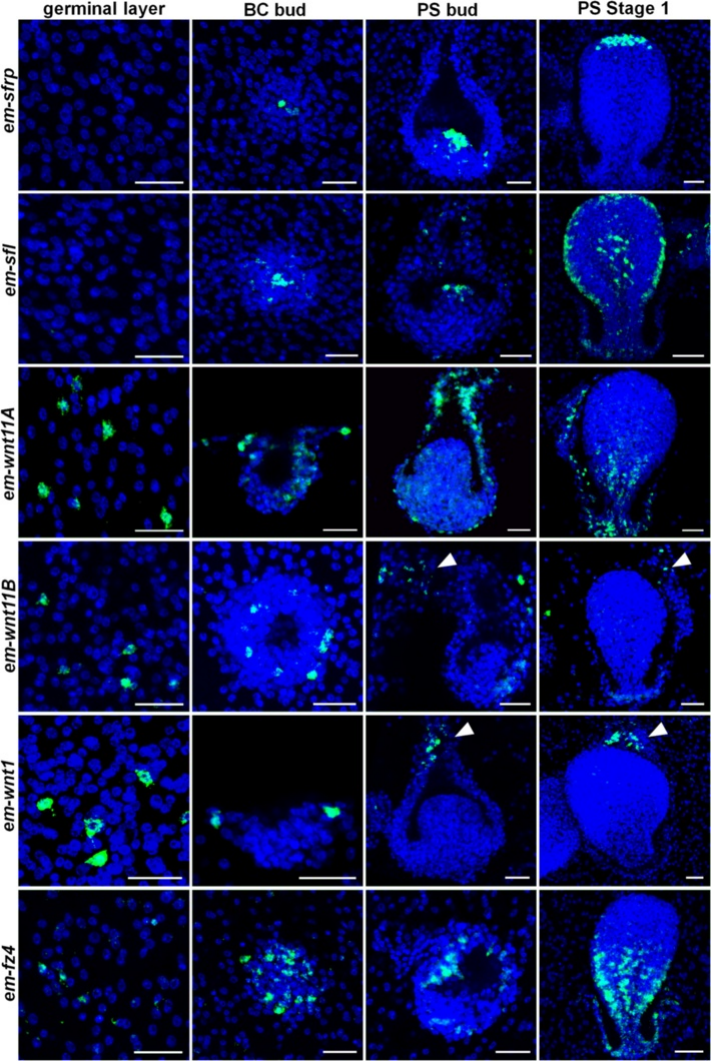
Expression of SFRP, posterior Wnt, and Frizzled receptor genes during early *E. multilocularis* protoscolex development. Whole-mount in situ hybridization signal is shown in green and nuclear DAPI staining in blue. GL: Germinal layer; BC bud: Early brood capsule bud (shown from above for all genes except for *em-wnt11A* and *em-wnt1*, for which it is shown from a side); PS bud: Early protoscolex bud; PS stage 1: First stage of protoscolex development following the system of Leducq and Gabrion [25]. Arrowheads indicate positive cells at the base of the brood capsule. Bars: 20 μm

**Fig. 3:**
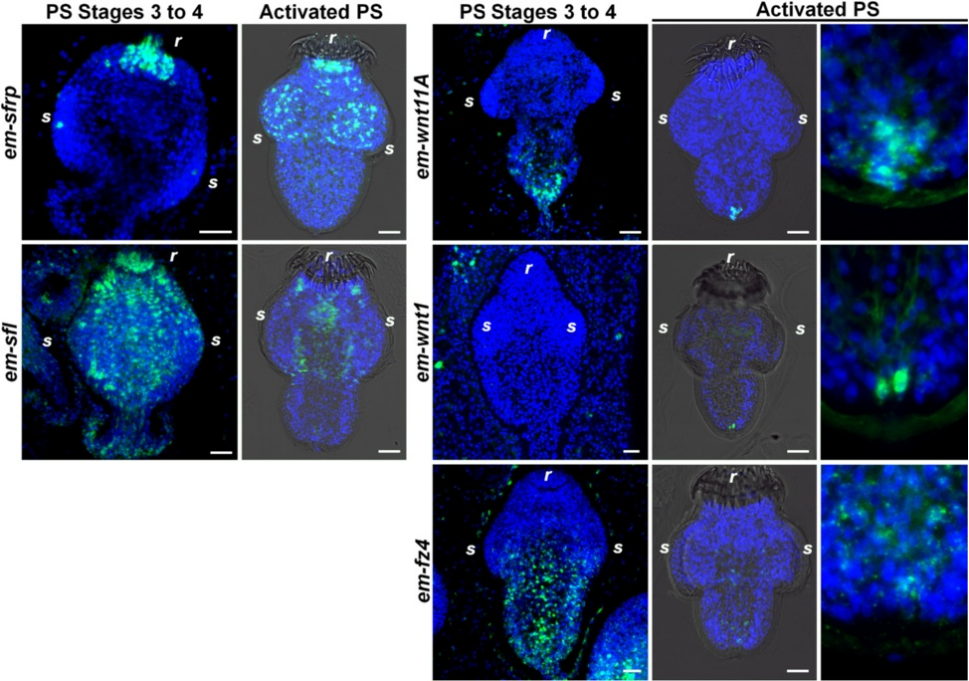
Expression of SFRP, posterior Wnt, and Frizzled receptor genes during late *E. multilocularis* protoscolex development. Whole-mount in situ hybridization signal is shown in green and nuclear DAPI staining in blue. PS stages 3 to 4: Stages 3 to 4 of protoscolex development following the system of Leducq and Gabrion [25]. Activated PS: Activated protoscoleces after isolation from metacestode vesicles and successive treatments with pepsin and sodium taurocholate. *s*, Suckers; *r*, Rostellum. Bars: 20 μm

We analyzed the expression of homologs of Wnt ligands and of inhibitors of Wnt signaling involved in planarian AP specification using whole-mount in situ hybridization (WMISH) [11, 12, 14, 15, 27] (Figs. 2 and 3). Although tapeworms have lost many developmental genes conserved in most animals, they possess clear homologs of all families of Wnt ligands present in planarians [28, 29]. They also have one clear member of the SFRP family of Wnt inhibitors containing a cysteine-rich domain and a netrin domain [28], and another member with a divergent netrin domain dubbed SFRP-like. In planarians, three different SFRP genes are expressed in overlapping anterior domains of which *sfrp-1* is considered a *bona fide* marker of anterior specification during regeneration [11, 14, 27, 30].

Strikingly, the *E. multilocularis em-sfrp* and *em-sfl* genes are not expressed in the germinal layer of the metacestode (they cannot even be detected by RT-PCR), and expression first appears when brood capsule buds develop as small accumulations of cells protruding from the germinal layer (Fig. 2). Throughout the development of the brood capsules and the protoscoleces, *em-sfrp* is expressed in the anterior-most region, eventually becoming restricted to a few cells at the tip of the developing protoscolex (Fig. 2). *em-sfl* also shows anterior expression but is less restricted, with strong expression in the apical end of the protoscolex but also in the protoscolex body and in the brood capsule (Fig. 2). These genes are the earliest known markers of brood capsule development. Conversely, when we analyzed the expression of homologs of posterior Wnt genes from planarians (*em-wnt1*, *em-wnt11A* and *em-wnt11B*), they were all expressed in dispersed cells in the germinal layer of the metacestode vesicles, and during brood capsule and protoscolex development they were always expressed in posterior domains: *em-wnt1* and *em-wnt11B* were always restricted to the germinal layer and to the base of the brood capsule, whereas *em-wnt11A* was expressed throughout the germinal layer and brood capsule, and also at the posterior end of the developing protoscolex (Fig. 2). These results are not only compatible with our hypothesis, but also suggest that the formation of brood capsules is induced by the specific inhibition of posterior Wnt ligands that are widespread in the germinal layer. They also correspond remarkably to expression patterns in planarians, in which SFRP and Wnt genes are expressed in anterior and posterior overlapping domains, respectively, with *wnt1* being the most posterior [12, 27].

**Fig. 4:**
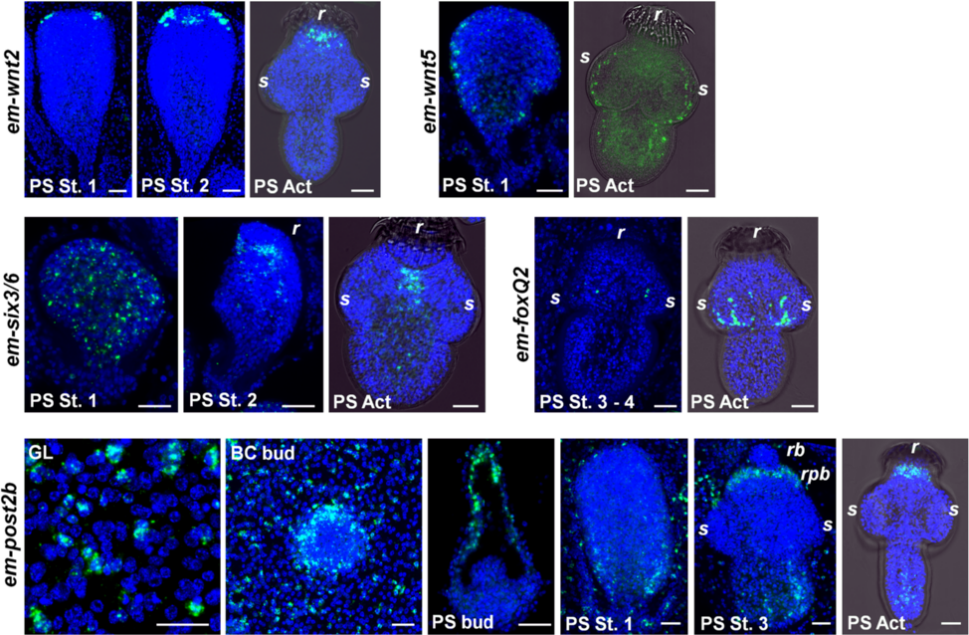
Expression of other Wnt genes and AP axis markers during *E. multilocularis* metacestode development. Whole-mount in situ hybridization signal is shown in green and nuclear DAPI staining in blue. PS Act, Activated protoscoleces; PS st 1 and 2: First and second stage of protoscolex development following the system of Leducq and Gabrion [25]; GL: Germinal layer; BC bud: Early brood capsule bud (shown from above for all genes except for em-wnt11A and em-wnt1, for which it is shown from a side); *rb*, Rostellar bulb; *rpb*, Rostellar pre-bulb; *s*, Suckers; *r*, Rostellum. Bars: 20 μm

Encouraged by these results, we analyzed the expression patterns of the remaining Wnt genes in *E. multilocularis* metacestodes (Fig. 4). The *wnt2* gene of planarians is expressed in two antero-lateral domains that surround the apical *sfrp*1^+^ cells [14, 27]. We see a remarkably similar expression pattern of *em-wnt2*, since it is not expressed at all in the germinal layer or brood capsules, and expression appears in two antero-lateral domains of the developing protoscolex which surround the *em-sfrp*^+^ expression domain. On the other hand, *wnt5* of planarians is expressed in lateral domains in planarians and is involved in the specification of the mediolateral axis through non-canonical Wnt signaling [27, 31]. Once again, we observe an equivalent pattern in the developing protoscolex, in which *em-wnt5* is expressed as two lateral stripes (Fig. 4). Unfortunately, we have not been able to obtain reproducible WMISH results for the remaining *wnt* gene, *em-wnt4*.

Finally, we analyzed the expression of *em-fz4*, an ortholog of a planarian Frizzled receptor expressed in the posterior-most region of the body and used as a specific posterior marker (named *fz4*, *fzT* or *fz-d* by different authors in different planarian species [11, 12, 32–34]) (Additional file 1). Frizzleds are a family of receptors for Wnt ligands that participate in both canonical and non-canonical signaling [35, 36]. The *em-fz4* gene is expressed in dispersed cells in the germinal layer, becomes strongly up-regulated during brood capsule development, and is always restricted to the posterior-most region of the protoscolex throughout development (Fig. 2).

### Expression patterns during later *E. multilocularis* protoscolex development

We also analyzed the expression of these genes during later protoscolex development, including completely developed protoscoleces, which had been activated by mimicking the infection of the definitive host. For some genes, the expression patterns did not vary significantly (for example, *em-wnt2* and *em-wnt5*; Fig. 3). For *em-wnt11a*, expression was restricted to the posterior region of the protoscolex throughout, becoming progressively more restricted and only being expressed in a few posterior-most cells in the developed protoscolex (Fig. 3). The *em-wnt1* and *em-wnt11b* genes are always restricted to the base of the brood capsule and are not expressed in the protoscolex itself (Fig. 3 and data not shown). Interestingly, after protoscolex release and activation, *em-wnt1* also becomes expressed in the posterior-most cells, similar to *em-wnt11a* (Fig. 3). Finally, *em-fz4* is always restricted to the posterior of the protoscolex and becomes barely detectable after development is complete (Fig. 3).

For other genes, the original expression patterns remained but additional sites of expression could be detected as development progressed. In the case of *em-sfrp*, the apical domain of expression persists throughout development, including in the apical rostellum (a muscular attachment organ containing hooks). However, during later development, new foci of expression appear in the scolex at each of the four developing sucker primordia, and expression remains in the suckers of the completely developed protoscolex (Fig. 3). In the case of e*m-sfl*, it was later expressed in many tissues during protoscolex development, but was restricted to the tissues surrounding the rostellum in the completely developed protoscolex (Fig. 3).

In summary, expression patterns observed during early developmental stages are shown to be largely maintained in later stages, but new expression domains appear that are not directly comparable to those of planarians. This is compatible with the hypothesis that early metacestode metamorphosis is the most highly conserved stage of development, and developmental gene expression diverges in later stages as tapeworm-specific characters, such as the attachment organs, are formed.

### Expression of conserved AP markers in *E. multilocularis*

In order to further test our hypothesis, we analyzed the expression patterns of AP markers that are well conserved in bilaterian animals but are not directly related to Wnt signaling. Our choices of markers were limited as many such genes have been lost in tapeworms or are too divergent to be identified unambiguously [29]. As a classical anterior maker, we have chosen the homeobox gene Six3/6 which is expressed in the anterior of bilaterian embryos (especially in the anterior-most region of the nervous system) [37]. In planarians, *six3/6* is expressed in the outer and anterior-most region of the brain [38, 39]. A clear ortholog of *six3/6* is present in tapeworms (Additional file 2). In *E. multilocularis*, *em-six3/6* is not expressed in the germinal layer. Low levels of expression first appear throughout the early protoscolex buds, and the expression domain of *em-six3/6* is progressively restricted during protoscolex development to the region behind the developing rostellum (Fig. 4). This area represents the rostellar nerve ring, the most anterior region of the central nervous system [24]. We also analyzed the expression of an ortholog of *foxQ2* (Additional file 3), which is expressed in the anterior-most region of many animals [40–42]. However, the planarian ortholog is expressed in the brain but not in the anterior-most region [39], and similarly, in *E. multilocularis em-foxQ2* appears to be expressed in the nervous system of the scolex, but expression only occurs during late development in the region where the nervous system associates with the suckers (postero-lateral ganglia and sucker nerve rings [24]; Fig. 4).

Hox genes have conserved roles in the specification of body regions along the AP axis in bilaterian animals [43]. As a classical posterior marker, we chose the posterior Hox gene *post2*, a homolog of which is also expressed in the posterior body of planarians [15, 44]. The *E. multilocularis* ortholog *em-post2b* [45] is strongly expressed in the germinal layer and brood capsule, and is restricted to the posterior most regions of the protoscolex during early development, thus supporting our hypothesis (Fig. 4). At later stages of protoscolex development, a second expression domain also appears in the rostellar pre-bulb region, which forms the rostellar hooks. This expression domain becomes dominant in the completely developed protoscolex (Fig. 4).

Therefore, once again, we observe comparable gene expression patterns in early metacestode development of *E. multilocularis* and in adults of planarians. During later metacestode development, divergent expression patterns appear, which are related to tapeworm-specific morphological innovations with no clear counterpart in planarians.

### Expression of Wnt and SFRP genes during *Hymenolepis microstoma* metamorphosis

Our results regarding gene expression in *E. multilocularis* strongly support our hypothesis that conserved gene expression patterns can be found during the larval metamorphosis of tapeworms. However, the development of *E. multilocularis* metacestodes is highly derived. Therefore, we wished to determine if similar gene expression patterns are also present in tapeworms with a more primitive form of development, and for this we chose the well-established model *H. microstoma* [46]. In *H. microstoma*, as in nearly all tapeworms, the oncosphere gives rise to a single juvenile worm with a scolex that develops at the pole opposite of the larval hooks (Fig. 1b). The polarity of the oncosphere is therefore reflected in the AP axis of the metacestode. However, like *E. multilocularis* and most other cyclophyllidean tapeworms, a primary lacuna (i.e. cavity) forms [47], later collapsing and encysting the nascent tapeworm.

**Fig. 5:**
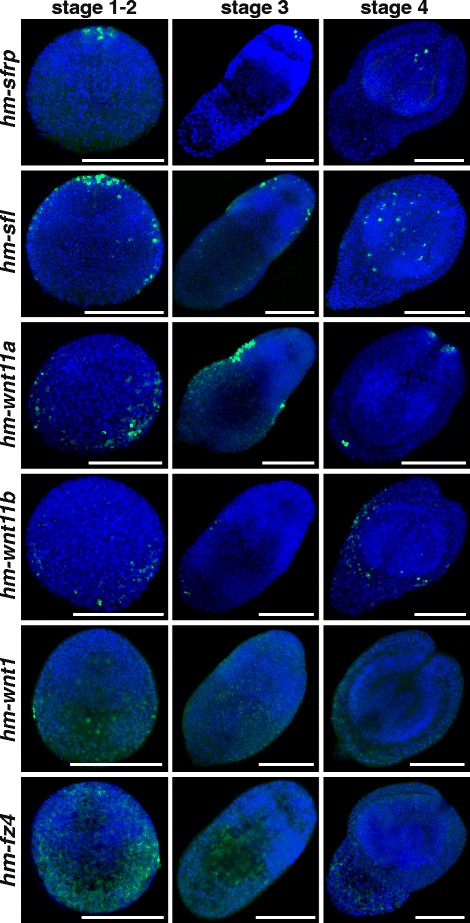
Expression of SFRP and ‘posterior’ Wnt genes during *H. microstoma* metacestode development. Staging of larvae follows Voge [47]. Whole-mount in situ hybridization signal is shown in green and nuclear DAPI staining in blue. Bars: 50 μm

**Fig. 6:**
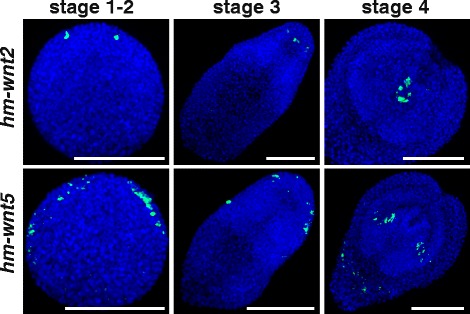
Expression of other Wnt genes during *H. microstoma* metacestode development. Staging of larvae follows Voge [47]. Whole-mount in situ hybridization signal is shown in green and nuclear DAPI staining in blue. Bars: 50 μm

Clear orthologs of all the described Wnt and SFRP genes of *E. multilocularis* are present in *H. microstoma*, and were identified with the same name, together with an *hm* prefix. As early as 48 hours after ingestion by the intermediate host, expression of the Wnt inhibitor *hm-sfrp* appears at the pole that will give rise to the scolex and is maintained throughout metamorphosis (Fig. 5). The *hm-sfl* gene is initially expressed apically, and later extends along the anterior region in two lateral stripes that begin sub-apically and end short of the opposite pole. In both cases, expression domains mirror those of *E. multilocularis* during protoscolex formation within the brood capsules (Fig. 2). Expression of ‘posterior’ Wnts *hm-wnt1*, *hm-wnt11a* and *hm-wnt11b*, and the frizzled receptor *hm-frzd4* is largely ubiquitous at the onset of metamorphosis, but becomes increasingly restricted to the posterior as the cells of the apical pole proliferate and condense to form the anterior regions. In addition, Wnts *hm-wnt11a* and *hm-wnt11b* show strong expression in two cells that mark the posterior pole, and *hm-wnt11a* also shows lateral expression in the central portion of the larvae (Fig. 5 and Additional file 4). Discrete expression of *hm-wnt11a* and *hm-wnt11b* at the posterior pole in *Hymenolepis* mirrors that seen in *E. multilocularis* during protoscolex development (Fig. 3). Thus, like in *E. multilocularis*, the metamorphosis of the *H. microstoma* larva begins with ubiquitous expression of Wnt ligands, from which a pole of Wnt inhibition leads to anterior development. The expression of genes coding for posterior Wnt ligands further supports the homology of the *E. multilocularis* germinal layer and the tissues that encyst the *Hymenolepis* juvenile. Other Wnt genes also replicate the expression domains observed in *E. multilocularis*: *hm-wnt2* is initially expressed in two anterolateral foci, which later expand slightly to surround the developing rostellum, whereas *hm-wnt5* is expressed in the lateral margins throughout larval development (Fig. 6 and Additional file 5). Taken together, the expression domains of these genes corroborate the expression of Wnt components seen in both tapeworm and planarian flatworms. They also help to confirm homologies between the convoluted morphology of *E. multilocularis* larvae and more typical tapeworm larval forms.

### Muscle cells are a source of Wnts in the *E. multilocularis* germinal layer

In planarians, Wnts, SFRPs, and other genes related to the specification of the body axes (the so-called ‘position control genes’, PCGs) are expressed by muscle cells in the body wall, providing positional information during normal tissue turnover and regeneration [48]. The products of PCGs influence the behavior of neoblasts, which are pluripotent stem cells that do not express PCGs.

**Fig. 7:**
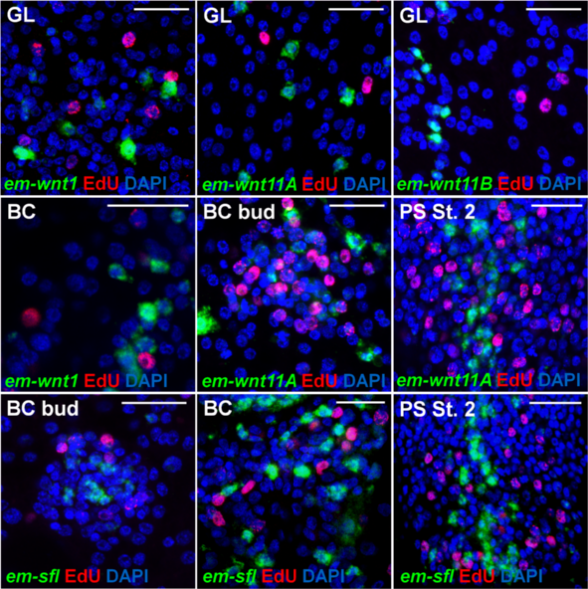
Lack of EdU incorporation in cells expressing Wnt and SFRP genes in *E. multilocularis* metacestodes. Green: Whole-mount in situ hybridization (WMISH); red: EdU detection; blue: nuclear DAPI staining. EdU labeling was done by culturing metacestodes *in vitro* for 5 hours with 50 μM EdU. Notice the lack of co-localization between WMISH and EdU for each gene at each stage. GL: Germinal layer; BC bud: Early brood capsule bud (shown from above for all genes except for em-wnt11A and em-wnt1, for which it is shown from a side); PS st 2: Second stage of protoscolex development following the system of Leducq and Gabrion [25]. Bars: 20 μm

In tapeworms, similar stem cells (often called germinative cells) exist [49]. We have recently characterized the stem cells of *E. multilocularis*, and demonstrated that these cells are the only proliferative cells, as is the case in all flatworms studied to date [50]. Therefore, they can be specifically labeled by incubation with thymidine analogs such as 5-ethynyl-2’-deoxyuridine (EdU), which are incorporated into DNA during replication [50]. In order to determine if Wnt and SFRP genes are expressed by stem cells, we performed double labeling of WMISH for each gene together with the detection of EdU incorporation. We observed little to no incorporation of EdU in cells positive for *em-wnt1* (0.0 %; n = 148 cells), *em-wnt11a* (2.2 %; n = 716 cells), and *em-wnt11b* (0.8 %; n = 241 cells) in the germinal layer (Fig. 7). This was also the case in developing brood capsules and protoscoleces for *em-wnt1* (0.3 %; n = 377 cells), *em-wnt11a* (1.6 %, n = 1,150 cells), *em-wnt11b* (0.7 %; n = 241 cells) and *em-sfl* (0.2 %; n = 426 cells) (Fig. 7). Furthermore, *em-sfrp* is only expressed in the post-mitotic apical region [50]. Therefore, as in planarians, there is no significant expression of PCGs in tapeworm stem cells.

**Fig. 8:**
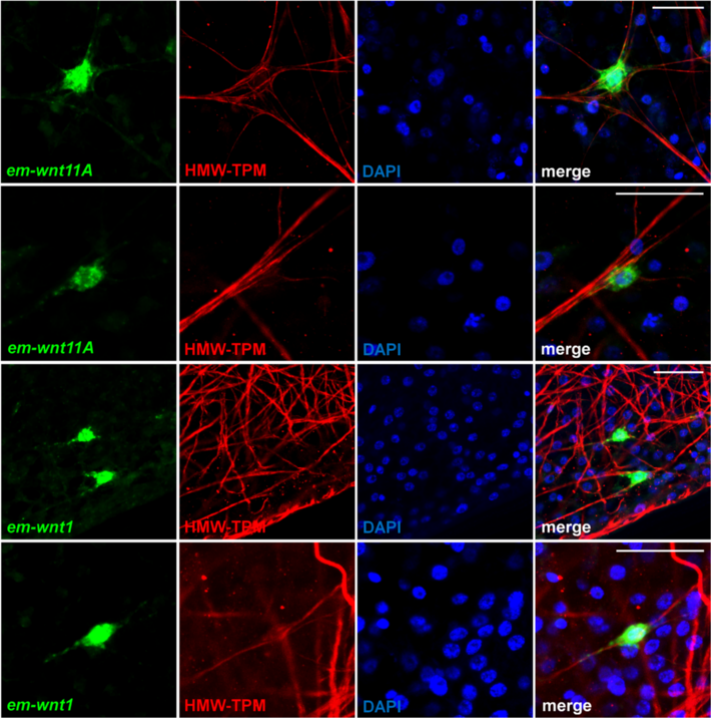
Expression of Wnt genes in muscle cells in *E. multilocularis* metacestode vesicles. Green: Whole-mount in situ hybridization; red: immunodetection of high molecular weight tropomyosins; blue: nuclear DAPI staining. For each gene, two examples of positive muscle cells with different morphologies are shown. Bars: 20 μm

In the germinal layer of *E. multilocularis* vesicles, there are muscle cells that form a layer of disorganized muscle fibers. In tapeworms, the contractile muscle fibers (myofibers) are only connected by thin cytoplasmic strands to the main cell body (myocyton) containing the nucleus and most organelles [51]. We performed immunohistofluorescence of metacestode vesicles with an anti-tropomyosin (TPM) antibody that specifically labels the muscle fibers of tapeworms, including those of *E. multilocularis* [50, 52, 53]. Besides the strong signal in large muscle fibers, in many cases we could also observe thin TPM^+^ filaments in the cytoplasm of myocytons that converged into myofibers (Fig. 8). By double labeling, we observed that many cells expressing *em-wnt1* and *em-wnt11a* in the germinal layer are muscle cells, since they also have TPM^+^ filaments in the surrounding cytoplasm, and the WMISH^+^ cytoplasm was clearly connected to long TPM^+^ myofibers (Fig. 8). Because not all muscle cells showed TPM^+^ fibers in the myocyton, we were unable to determine the percentage of muscle cells expressing each gene. Therefore, posterior Wnts are not only expressed in similar domains in *E. multilocularis* metacestodes and planarians adults, but also by the same cell type.

## Discussion

### The polarity of the tapeworm AP axis is conserved with other bilaterians

Our results clearly support the classical assumption that the scolex represents the true anterior end of tapeworms, and suggest that the condensation of the nervous and osmoregulatory systems present in the scolex are examples of cephalization. One of the main historical controversies regarding the AP axis of cestodes has been the interpretation of the polarity of the oncosphere, and how it relates to the polarity of the metacestode and adult [8]. The pole containing the hooks and the pores of the penetration glands has been considered functionally anterior, but the scolex develops from the opposite pole. However, oncospheres lack a brain (even the presence of nerve cells is controversial), do not move directionally and are taken up passively by the host [8, 54]. The most basal cestode lineages (Amphilinidea and Gyrocotylidea) have a more complex ciliated larva with ten hooks called the lycophora. This larva has an anterior brain and penetrates actively through the body wall of the intermediate host [55]. Importantly, during swimming the end of the lycophora bearing the hooks is directed posteriorly [56]. In the amphilinidean *Austramphilina elongata*, once the lycophora larva makes contact with the host with its anterior end, it bends into a U-shape that brings the posterior hooks and the anterior end together as the hooks participate in the penetration of the host [55]. During development of the juvenile, the end opposite of the hooks develops into a sucker for attachment [55]. Therefore, in basal cestodes, the functional end of the larva is the same as that of the adult, and the hooks are simply posterior organs for attachment and penetration. Wnt expression corroborates this notion and thus supports an evolutionary transition in which the oncosphere was derived by extensive reduction of a lycophora larva, and the posteriorly-oriented hooks maintained their role for penetration (now through the gut of the host) as eucestodes transitioned to a passive mode of infection of the intermediate host.

Another classical argument opposing the view that the scolex is the anterior pole of tapeworms is the fact that, if the scolex is considered anterior, then the relative position of the male and female gonads (testes anterior to the ovary) would be opposite that found in free-living flatworms. It is therefore possible that a change in the relative position of the gonads in the adult (that is, after the developmental stages covered in this work) occurred during tapeworm evolution. Finally, when the scolex is considered anterior, tapeworms show a form of growth that is contrary to that of all other segmented animals, as new segments are formed in the anterior germinative region in the neck (as opposed to a posterior growth zone as seen, for example, in annelids) [10]. However, tapeworm segmentation evolved *de novo* in the eucestode lineage and is not homologous to that seen in other animals [3, 57], making developmental comparisons impossible.

### A phylotypic stage for flatworms

The Neodermata and several derived groups of free-living flatworms have extremely divergent modes of early embryonic development as a consequence of their unique eggs, which contain not only the oocyte, but also specialized cells called vitellocytes that produce yolk and shell proteins [2, 58]. Different flatworm groups have developed independent solutions to the problem of incorporating the yolk mass into the embryo, resulting in unique early developmental patterns. This is in stark contrast to the spiral cleavage present in most basal flatworm lineages and also in many other members of the superphylum Lophotrochozoa [58]. Therefore, it is very difficult to search for homologies in the development of different flatworm groups based solely on morphological grounds.

**Fig. 9:**
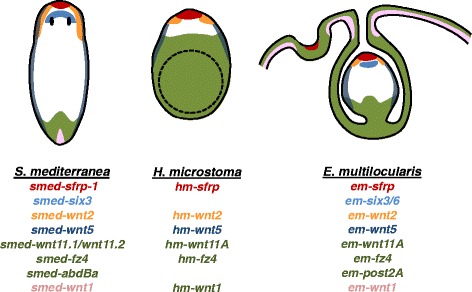
Comparison of gene expression patterns in adults of the planarian *Schmidtea mediterranea*, with *H. microstoma* and *E. multilocularis* metacestodes. Approximate expression domains are shown with different colors, and in some cases several genes with slightly different expression domains were grouped together. References for the *S. mediterranea* expression patterns are given in the main text

Despite this morphological diversity, we observe that the expression patterns of genes related to AP polarity and other PCGs are strikingly similar in tapeworms during larval metamorphosis and in planarians at late stages of embryonic development (Fig. 9). In contrast, early embryonic stages are highly divergent, and represent specific adaptations to their unique lifestyles. In planarians, early blastomeres disperse into the mass of vitellocytes (in a process aptly named ‘blastomere anarchy’) and eventually surround it. Later, a transient embryonic pharynx is formed [16]. All of these modifications are a consequence of the need to incorporate the external yolk, arising as an evolutionary adaptation to competition between several embryos within a single egg for limited yolk resources [16, 58]. During early development of planarians, many PCGs are already expressed, but in patterns that do not resemble those in later stages or in other animals, and the relationships between the axes of the early embryo and the adult are not clear [59]. At later stages of development, the definitive regions and tissues of the body of the embryo arise from ventral condensations of blastomeres, and it is at this stage that the PCGs acquire canonical expression patterns [59]. A similar developmental stage has been described in other flatworm groups, and it has been proposed to be the phylotypic stage for free-living flatworms [58]. The phylotypic stage is usually defined as a stage during mid-embryogenesis where the similarity in body plan and corresponding expression of developmental genes is maximal across the species of a phylum, and the definitive body plan of the animal is established [60, 61]. The phylotypic stage is expected to be highly conserved due to developmental constraints of numerous global interactions (unlike early development, which is highly variable in many taxa) and significant similarities can also be drawn at this stage with gene expression patterns in other animal phyla [60, 61].

We propose that the phylotypic stage suggested for free-living flatworms also applies to tapeworms. In the case of tapeworms, early development is also divergent, and the formation of the oncosphere is an evolutionary adaptation for the infection of the first host of the life cycle. The metacestode stage can be considered to have the basic body plan of tapeworms, as in basal cestode groups the only further development that occurs is sexual maturation, and segmentation in eucestodes is an evolutionarily derived novelty imposed over this basic body plan [3, 55, 57]. Therefore, during early metamorphosis of the oncosphere, the body plan is established and it is precisely at this stage that strong conservation is seen in the expression patterns of metazoan developmental genes. Later, development again diverges between groups, as specific organs of attachment are formed, representing the onset of the next ontogenetic phase of the life cycle. The expression of some PCGs diverges at this point, suggesting that they have become exapted for novel roles, in agreement with the idea that only the early metamorphosis represents the phylotypic stage. It would be interesting to determine the expression of PCGs in early tapeworm embryogenesis, but this is technically challenging as the embryo develops within the egg, and in cyclophyllideans also *in utero.*

In planarians, the anterior pole of the head has been proposed to organize anterior development during adult regeneration and expresses *sfrp-1* together with other inhibitors of Wnt signaling [62]. An apical pole of *sfrp-1* expression is also the earliest expression pattern of a PCG in the embryo that resembles that found in the adult [59]. Similarly, an apical pole of *sfrp* expression is observed very early during tapeworm anterior development. Interestingly, this region later becomes the rostellum, and continues to express *sfrp*. The rostellum is an evolutionary modification of the apical organ, an attachment organ that is formed in all tapeworms during metamorphosis and which may persist in the adult or be only transitory [4, 18]. Because of the universal conservation of this apical organ in tapeworms, it is possible that it also has a conserved role as an anterior organizer and, in fact, such a role has been previously proposed for the rostellum [63].

### Evolutionary origins of the unique *E. multilocularis* development

The massive metacestodes of *Echinococcus* spp. result from extensive growth of the germinal layer. In the related metacestodes of *Taenia* spp. (that together with *Echinococcus* spp. comprise the family Taeniidae), the bladder also reaches large proportions, and one or many scoleces develop from the bladder tissue. The morphology of the metacestodes from *Taenia* spp. can be remarkably varied, including large bladders with a single scolex (cysticercus), with many invaginated scoleces (coenurus), or with many externally protruding scoleces (‘polycephalic larvae’) [17, 64, 65], but in all cases the scolex develops towards the exterior of the bladder (exogenous development). Asexual formation of many scoleces is found in many basal taeniid lineages, suggesting that it was present in the last common ancestor of the family, but homology of asexual reproduction in taeniids is controversial [65–69]. Because the development of *E. multilocularis* is actually also exogenous, we propose that there is no fundamental difference in the development of taeniid metacestodes, supporting the hypothesis that asexual scolex formation is ancestral in taeniids (and was lost secondarily in several lineages). One unique characteristic of *E. multilocularis* metacestodes is the secretion of a thick external acellular covering, the laminated layer [70]. The brood capsules form as invaginations of the germinal layer that are not accompanied by the laminated layer. Therefore, brood capsules can be regarded as specializations of the germinal layer in which protoscoleces can develop without a laminated layer cover.

The *E. multilocularis* metacestode initially forms as a hollow cyst that lacks morphological polarity, and shows widespread expression of posterior Wnt genes. This suggests that, in *E. multilocularis*, anterior development is suppressed during the early stages of the oncosphere-to-metacestode transition, and that Wnt signaling is important for maintaining the posterior identity of the tissues. The formation of protoscoleces only occurs after months of infection [21, 71], and is accompanied by the expression of Wnt inhibitors, indicating that suppression of Wnt signaling is important for the formation of anterior structures. In contrast, in other tapeworms, the scolex primordium develops very early (for example, at 48 hours post-infection in *H. microstoma*) as a condensation of cells that always forms opposite the oncospheral hooks [8]. In classical studies of *E. multilocularis* metamorphosis, there is no indication of early anterior development and later regression, suggesting that anterior development is suppressed from the beginning [21]. Scattered studies of the development of metacestodes of the related *Taenia* spp. also indicate that they initially lack AP polarity, and develop as bladders from which one or more scolex primordia appear later [63, 72–76]. The lack of AP polarity during initial metacestode development, leading to a lack of correspondence between the polarity of the oncosphere and that of the metacestode, may be a characteristic of all taeniids.

Finally, the presence of muscle fibers in *E. multilocularis* metacestode vesicles has been enigmatic, since the fibers are disorganized and the vesicles lack motility [21, 24, 77]. The muscle system is probably homologous to that found in the bladder of other taeniids: in *Taenia* spp., the bladder tissue also has muscle fibers, is motile, and may assist in the evagination of the scolex [17, 24, 78]. Herein, we show that *E. multilocularis* muscle cells are a source of Wnt ligands, and suggest that this may account for the retention of this cell type in the otherwise immobile metacestode.

## Conclusions

The unique biology of tapeworms has been a source of fascination and speculation, but their uniqueness has also confounded our ability to compare them to other animals and has hampered our understanding of their evolutionary origins. Identification of a conserved developmental stage in tapeworms opens the possibility to perform meaningful comparisons with free-living flatworms and other phyla for the first time. Conservation of the flatworm stem cell system and of underlying gene regulatory networks suggests that reciprocal illumination will come from studies of both planarians and parasitic flatworms [79].

## Methods

### Maintenance, culture and collection of tapeworms

*E. multilocularis* isolates were maintained by serial intraperitoneal passage in *Meriones unguiculatus* as previously described [80]. The isolates used were GH09 and J2012, obtained from accidental infections of Old World Monkeys in a breeding enclosure [81], and MS10/10, obtained from an infected dog. All isolates had been passaged for 5 years or less at the time of this study. Animal experiments were carried out in accordance with European and German regulations on the protection of animals (Tierschutzgesetz). Metacestode vesicles obtained from *in vivo* culture were co-cultured *in vitro* with rat Reuber hepatoma cells as previously described [80]. After at least 2 months of *in vitro* culture, metacestode vesicles began the development of brood capsules and protoscoleces, and were collected and fixed for WMISH and immunohistofluorescence with 4 % paraformaldehyde prepared in phosphate buffered saline following the method of Koziol et al. [50]. Completely developed protoscoleces were isolated from parasite material maintained *in vivo*, and activated by successive treatments with pepsin at low pH and sodium taurocholate [82].

The Nottingham strain of *H. microstoma* [46] was maintained *in vivo* using outbred BALB/c mice and flour beetles (*Tribolium confusum*). Infection of beetles was produced by exposing them for 24 hours to macerated gravid tissues of *H. microstoma*. Then, beetles were allowed to feed on flour and metacestodes at different stages of metamorphosis were obtained by dissection on successive days. Larvae were fixed with paraformaldehyde prepared in phosphate buffered saline, as previously described [50].

### Molecular cloning

Genes of interest were identified by BLAST searches against the genomes and derived gene models of *E. multilocularis* and *H. microstoma* [29] followed by phylogenetic analyses (Additional files 1, 2 and 3). Fragments of the coding domain sequences of *E. multilocularis* genes were amplified by RT-PCR using pools of cDNA from *in vitro* cultured metacestode vesicles, primary cells and protoscoleces, and cloned into pDrive (Qiagen, Hilden, Germany) or pJet1.2 (Thermo Scientific, Schwerte, Germany) following standard procedures, as previously described [50]. *Hymenolepis* transcripts were amplified using cDNA from larval or adult specimens and cloned into StrataClone vectors (Agilent Technologies, La Jolla, CA, USA). Colonies were picked and checked for insert directionality via PCR, then used as templates for PCR amplification using M13 primers, resulting in products with T3/T7 promoter sites that were purified and used subsequently as templates for reverse transcription. A list of all gene model codes and primers used in this work is provided in Additional file 6.

### Whole-mount in situ hybridization (WMISH)

Tyramide-FITC-based fluorescent and alkaline phosphatase-based conventional WMISH of *E. multilocularis* and *H. microstoma* larval stages were performed with digoxigenin-labeled antisense probes, as previously described [50]. *E. multilocularis* samples were analyzed by confocal microscopy using a Leica TCS SP5 (Leica Microsystems, Wetzlar, Germany). All gene expression patterns reported in this work from *E. multilocularis* metacestodes were obtained from at least two different WMISH experiments, starting from independent batches of *in vitro* cultured animals, with at least five metacestode vesicles analyzed in each experiment. Those of *Hymenolepis* are based on multiple independent assays, each containing approximately 10 *in vivo*-reared specimens of each larval stage (approximately 50 larvae per tube). Alkaline phosphatase-based WMISH specimens were imaged using differential interference contrast microscopy on a Leica DM5000B light microscope. Fluorescent specimens were imaged using a Nikon A1 confocal microscope and maximum projections created using ImageJ v. 2 [83]. Control experiments using labeled sense probes were always negative (data not shown).

### *In vitro* labeling with 5-ethynyl-2’-deoxyuridine (EdU)

*In vitro* labeling with 50 μM EdU was done for 5 hours and fluorescent detection with Alexa Fluor 555 azide was performed after WMISH following the method described in by Koziol et al. [50].

### Immunohistofluorescence

The immunohistofluorescence procedure described by Koziol et al. [24] was carried out after WMISH detection. Anti-HMW-tropomyosin [53] was used as primary antibody at a 1:500 dilution. The secondary antibody was anti-rabbit conjugated to tetramethylrhodamine (Jackson ImmunoResearch, West Grove, PA, USA).

## Additional files

Additional file 1:
**Phylogeny of Frizzled receptors.** Frizzled receptors from *H. sapiens*, *D. melanogaster*, the trematode *S. mansoni* [84], the planarian *S. mediterranea* [84], and the tapeworms *E. multilocularis* and *H. microstoma* [29] were aligned, and a phylogeny was estimated by Maximum Likelihood analysis (with a JTT model) using MEGA 5.2 [85]. The tree was rooted using the related Smoothened receptors. Bootstrap support values from 1,000 replicates are indicated next to the nodes. Nodes with lower than 50 % support were collapsed. Genbank accession codes are given for *H. sapiens* and *D. melanogaster*; GeneDB accession codes are given for *E. multilocularis* and *H. microstoma*; gene names from [84] are given for *S. mansoni* and *S. mediterranea*. The *fz4* genes of *E. multilocularis* and *H. microstoma* are indicated by arrows. (PDF 9 kb) Additional file 2:
**Phylogeny of sine oculis-homeobox (SIX-HD) transcription factors.** The homeodomain (HD) and SIX domains of SIX-HD proteins of *H. sapiens*, *D. melanogaster*, *E. multilocularis*, and *H. microstoma* were aligned together with published planarian SIX-HD proteins, and a phylogeny was estimated by Maximum Likelihood analysis (with a JTT model) using MEGA 5.2 [85]. Bootstrap support values from 1,000 replicates are indicated next to the nodes. Genbank accession codes are given for *H. sapiens*, *D. melanogaster*, and for the planarians *S. mediterranea* and *G. tigrina*; GeneDB accession codes are given for *E. multilocularis* and *H. microstoma.* Different groups of SIX-HD proteins are outlined, and the *six3/6* genes of *E. multilocularis* and *H. microstoma* are indicated by arrows. (PDF 109 kb) Additional file 3:
**Phylogeny of Forkhead (FOX) transcription factors.** The FKH domain of forkhead proteins of *H. sapiens*, *D. melanogaster*, *E. multilocularis*, *H. microstoma*, and published FoxQ2 homologs from other animals were aligned, and a phylogeny was estimated by Maximum Likelihood analysis (with a JTT model) using MEGA 5.2 [85]. Bootstrap support values from 1,000 replicates are indicated next to the nodes. Nodes with lower than 50 % support were collapsed. GeneDB accession codes are given for *E. multilocularis* and *H. microstoma* and Genbank accession codes are given for all other sequences. The FoxQ2 group is outlined, and the *foxQ2* genes of *E. multilocularis* and *H. microstoma* are indicated by arrows. (PDF 33 kb) Additional file 4:
**Alkaline phosphatase-based development of whole-mount in situ hybridization in** ***H. microstoma*** **: SFRPs and posterior Wnt components.** Bars: 50 μm. (PDF 5034 kb) Additional file 5:
**Alkaline phosphatase-based development of whole-mount in situ hybridization in** ***H. microstoma*** **: Wnt2 and Wnt5.** Bars: 50 μm. (PDF 1653 kb) Additional file 6:
**List of genes studied in this work.** The GeneDB codes (www.genedb.org) of *E. multilocularis* and *H. microstoma* gene models are given, together with the primer sequences used for transcript amplification by RT-PCR. (XLSX 12 kb)

## Abbreviations

AP: Antero-posterior
EdU: 5-ethynyl-2’-deoxyuridine
PCG: Position control gene
TPM: Tropomyosin
WMISH: Whole-mount in situ hybridization
