# Supplementary figures and images for: Comparative analysis of Wnt expression identifies a highly conserved developmental transition in flatworms
Source: BMC Biol. 2016 Mar 4;14:10. doi: 10.1186/s12915-016-0233-x (PMC4778295; doi:10.1186/s12915-016-0233-x)

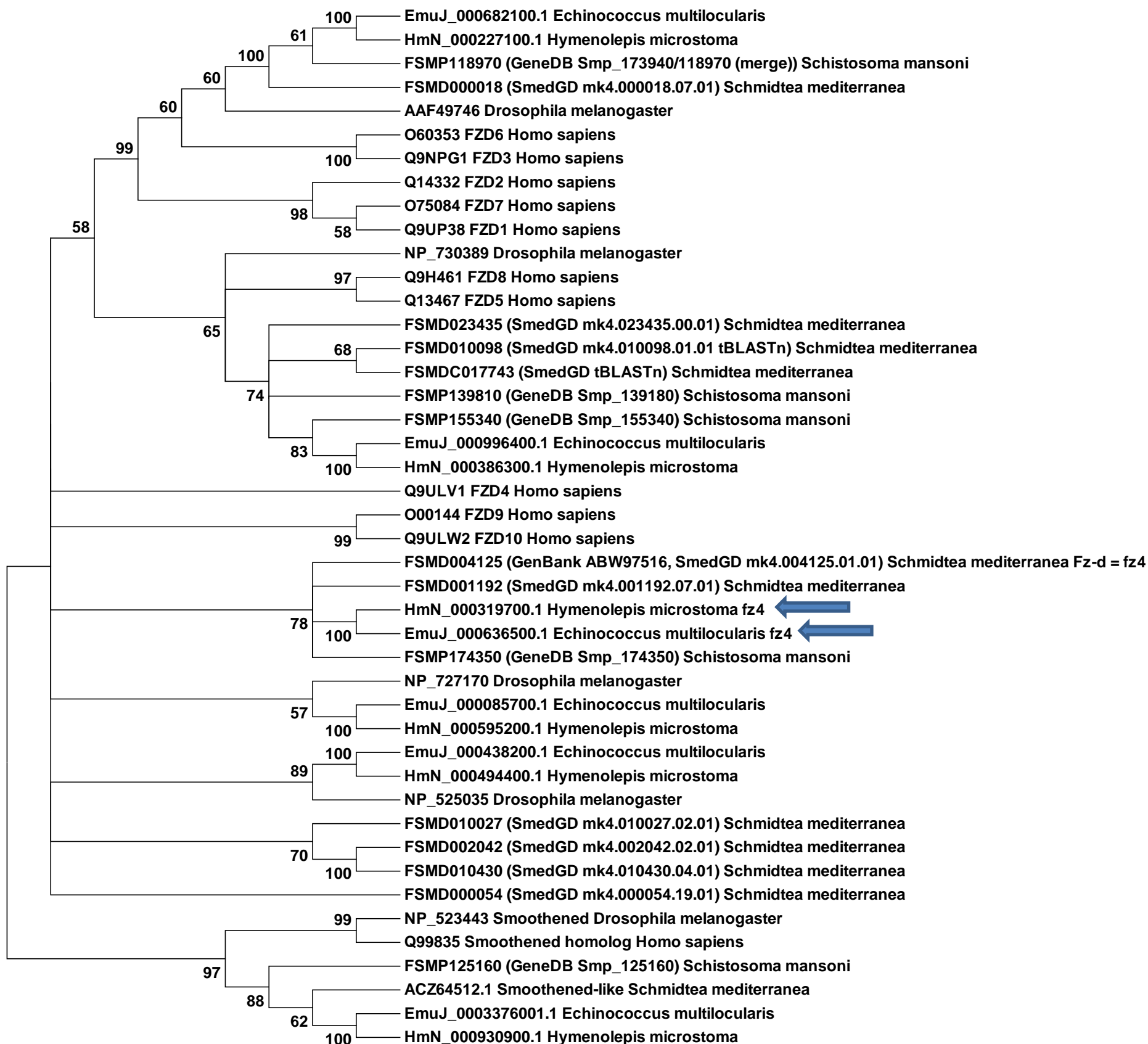

Supplement: Additional file 1: — Phylogeny of Frizzled receptors. Frizzled receptors from H. sapiens, D. melanogaster, the trematode S. mansoni [84], the planarian S. mediterranea [84], and the tapeworms E. multilocularis and H. microstoma [29] were aligned, and a phylogeny was estimated by Maximum Likelihood analysis (with a JTT model) using MEGA 5.2 [85]. The tree was rooted using the related Smoothened receptors. Bootstrap support values from 1,000 replicates are indicated next to the nodes. Nodes with lower than 50 % support were collapsed. Genbank accession codes are given for H. sapiens and D. melanogaster; GeneDB accession codes are given for E. multilocularis and H. microstoma; gene names from [84] are given for S. mansoni and S. mediterranea. The fz4 genes of E. multilocularis and H. microstoma are indicated by arrows. (PDF 9 kb) [file 12915_2016_233_MOESM1_ESM.pdf]

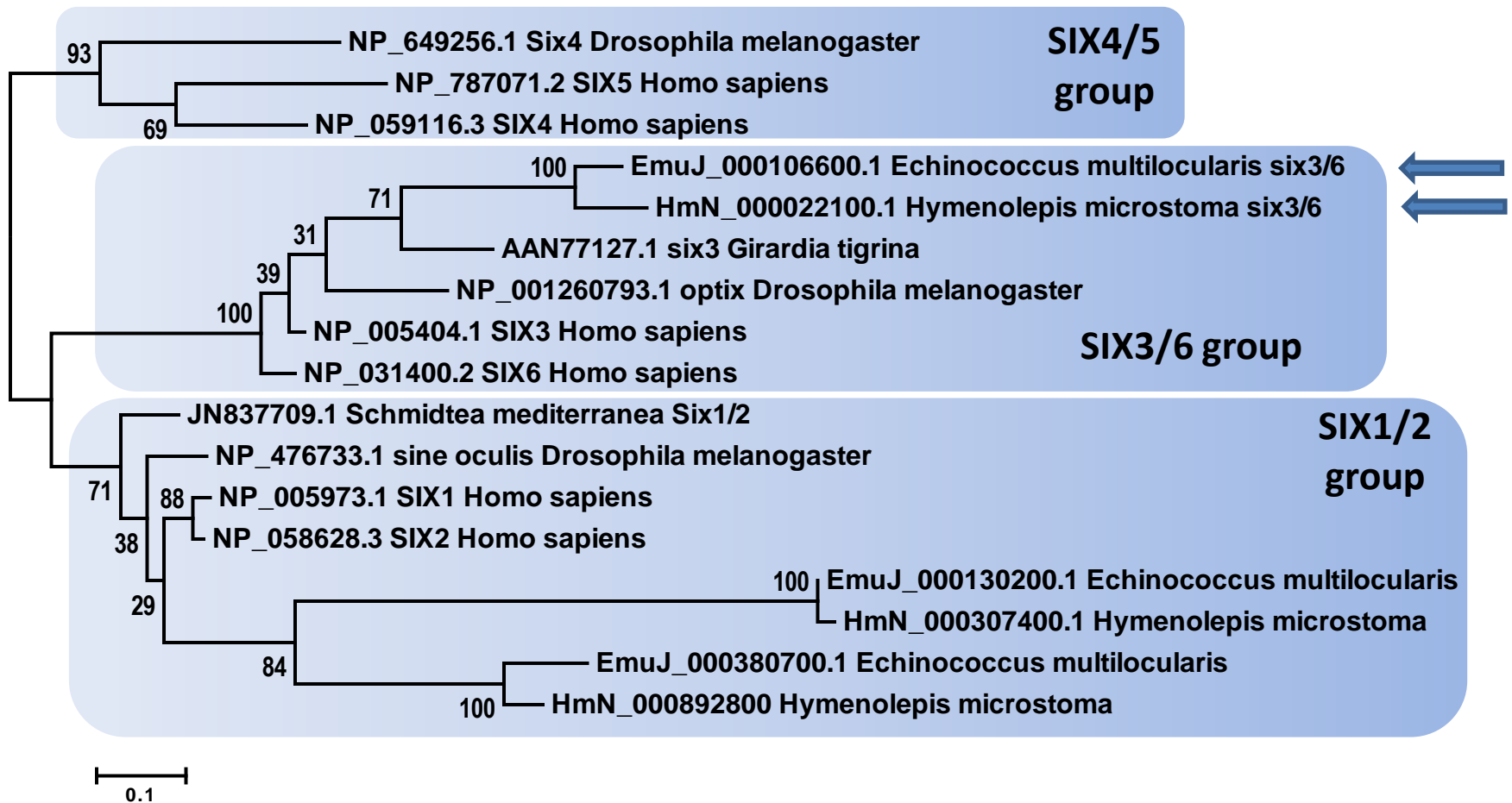

Supplement: Additional file 2: — Phylogeny of sine oculis-homeobox (SIX-HD) transcription factors. The homeodomain (HD) and SIX domains of SIX-HD proteins of H. sapiens, D. melanogaster, E. multilocularis, and H. microstoma were aligned together with published planarian SIX-HD proteins, and a phylogeny was estimated by Maximum Likelihood analysis (with a JTT model) using MEGA 5.2 [85]. Bootstrap support values from 1,000 replicates are indicated next to the nodes. Genbank accession codes are given for H. sapiens, D. melanogaster, and for the planarians S. mediterranea and G. tigrina; GeneDB accession codes are given for E. multilocularis and H. microstoma. Different groups of SIX-HD proteins are outlined, and the six3/6 genes of E. multilocularis and H. microstoma are indicated by arrows. (PDF 109 kb) [file 12915_2016_233_MOESM2_ESM.pdf]

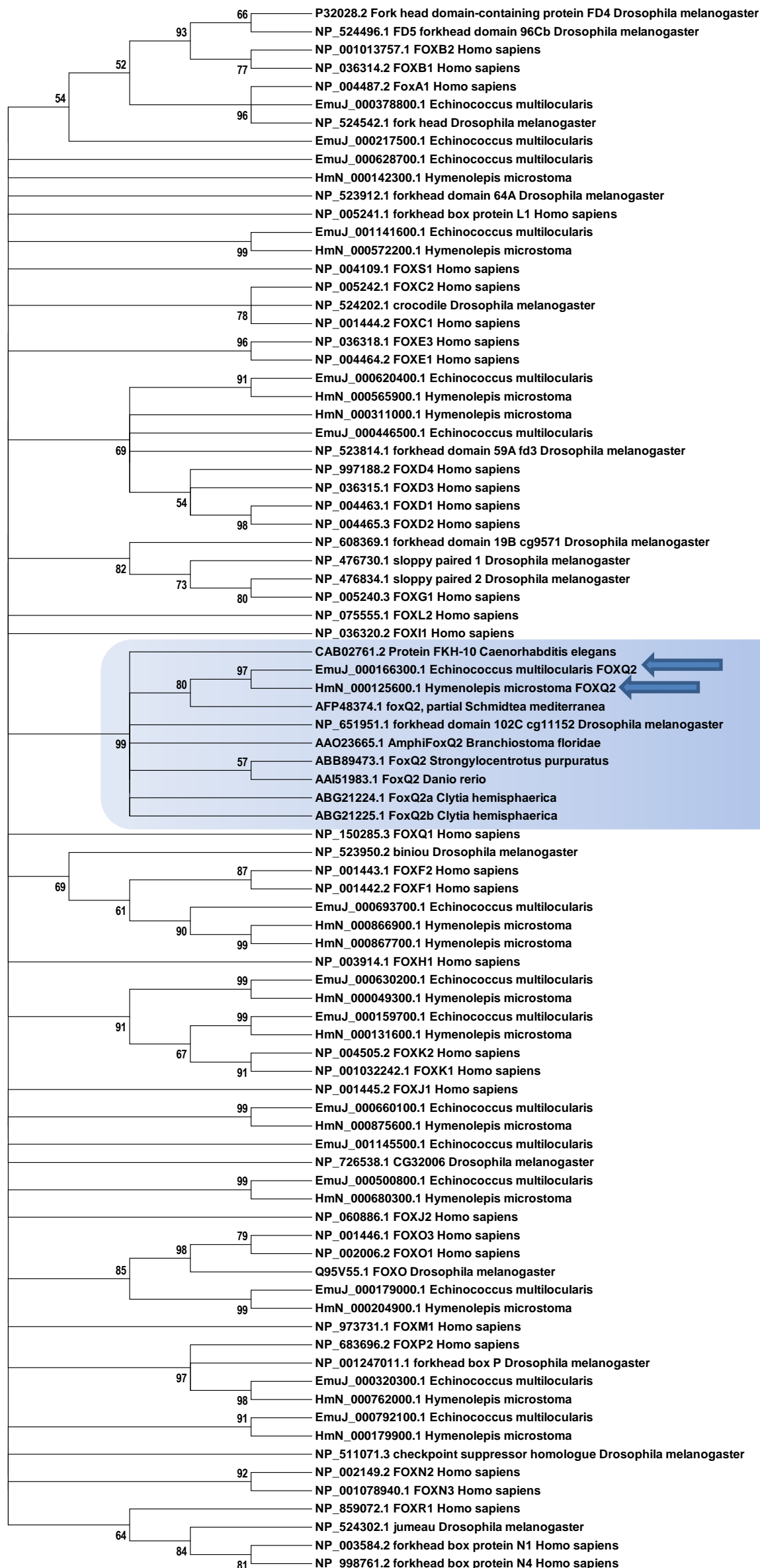

**FoxQ2  
group**

Supplement: Additional file 3: — Phylogeny of Forkhead (FOX) transcription factors. The FKH domain of forkhead proteins of H. sapiens, D. melanogaster, E. multilocularis, H. microstoma, and published FoxQ2 homologs from other animals were aligned, and a phylogeny was estimated by Maximum Likelihood analysis (with a JTT model) using MEGA 5.2 [85]. Bootstrap support values from 1,000 replicates are indicated next to the nodes. Nodes with lower than 50 % support were collapsed. GeneDB accession codes are given for E. multilocularis and H. microstoma and Genbank accession codes are given for all other sequences. The FoxQ2 group is outlined, and the foxQ2 genes of E. multilocularis and H. microstoma are indicated by arrows. (PDF 33 kb) [file 12915_2016_233_MOESM3_ESM.pdf]

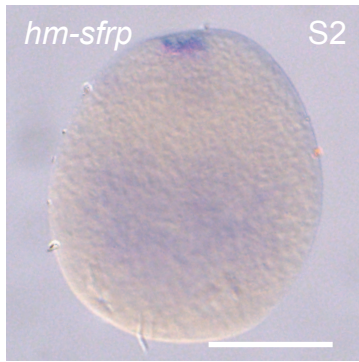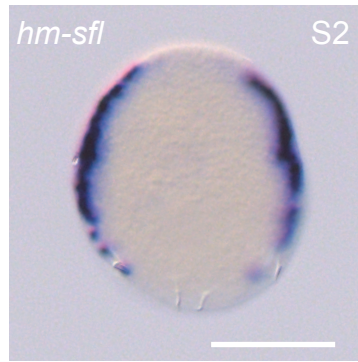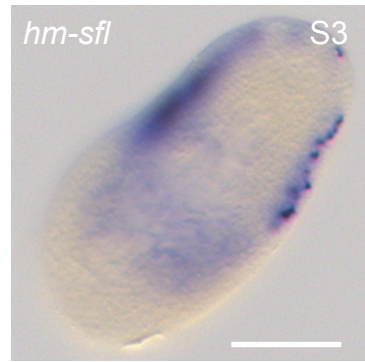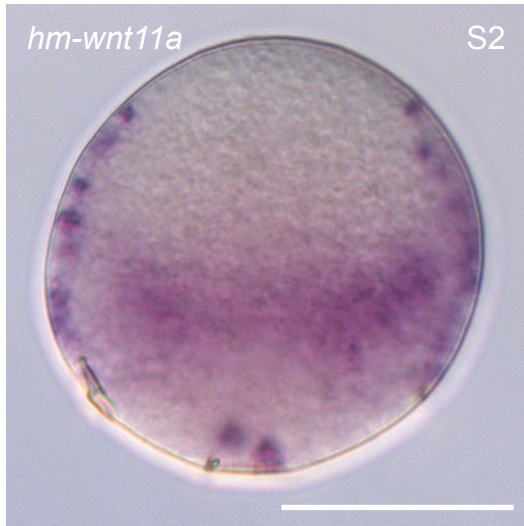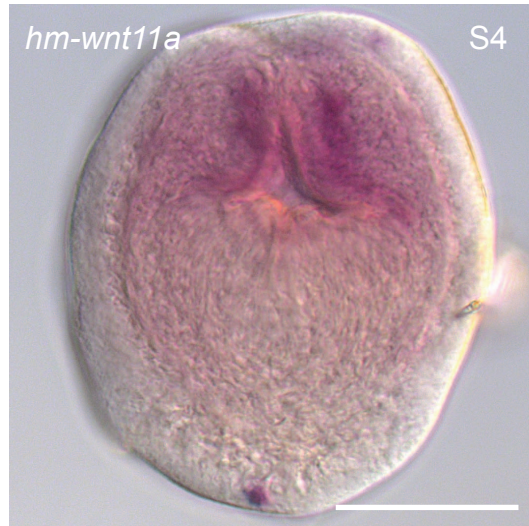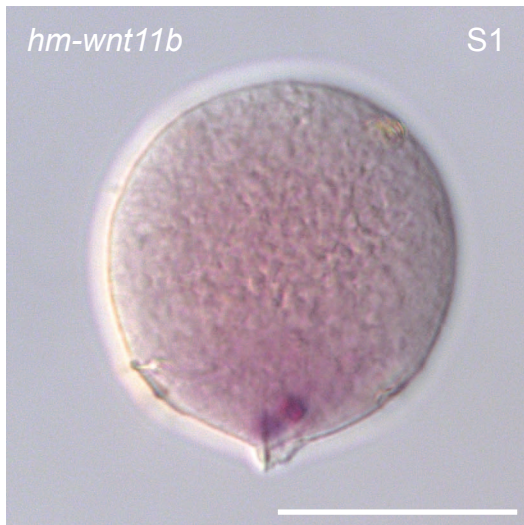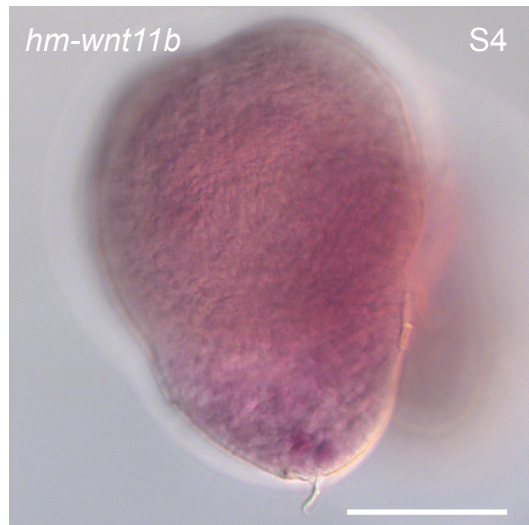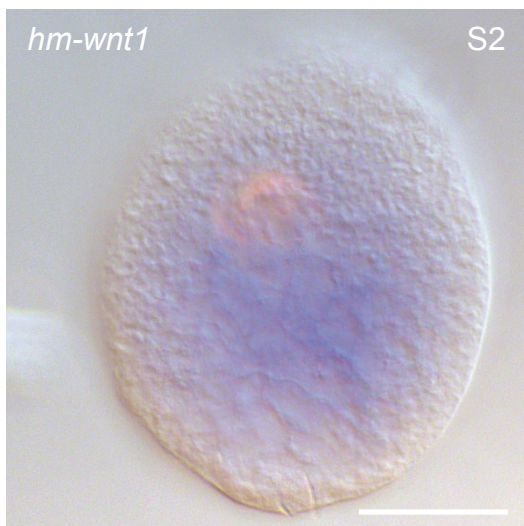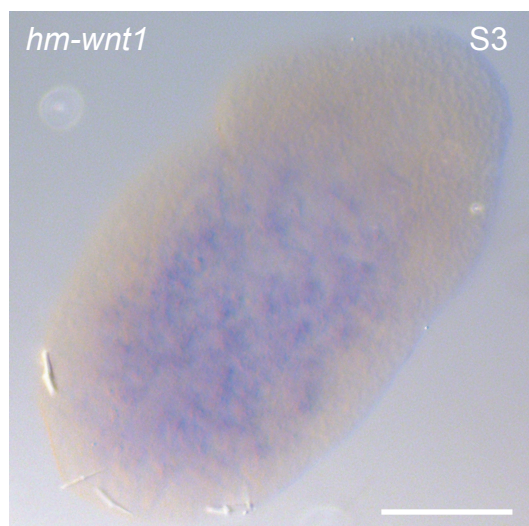

Supplement: Additional file 4: — Alkaline phosphatase-based development of whole-mount in situ hybridization in H. microstoma : SFRPs and posterior Wnt components. Bars: 50 μm. (PDF 5034 kb) [file 12915_2016_233_MOESM4_ESM.pdf]

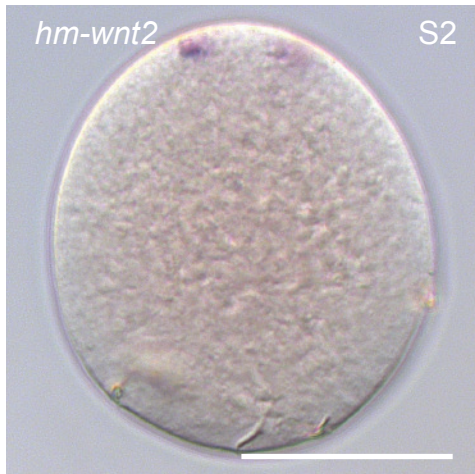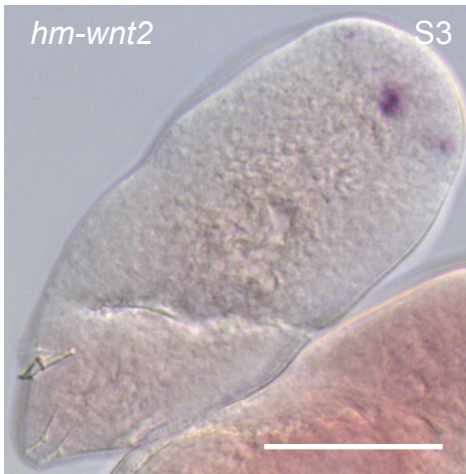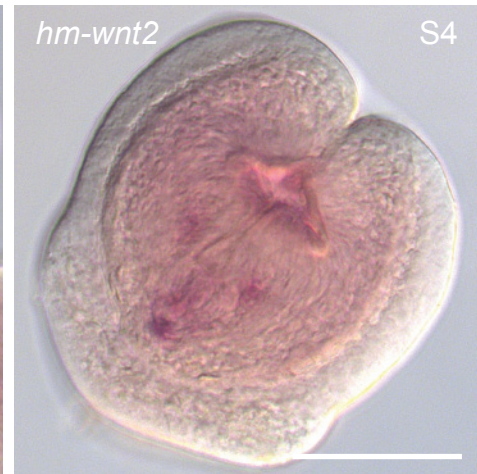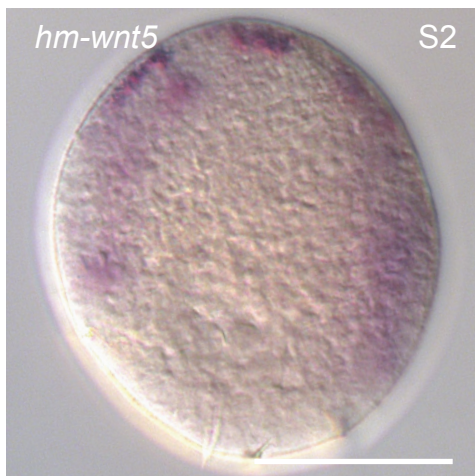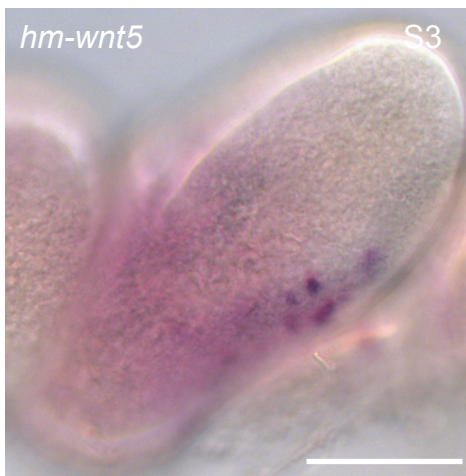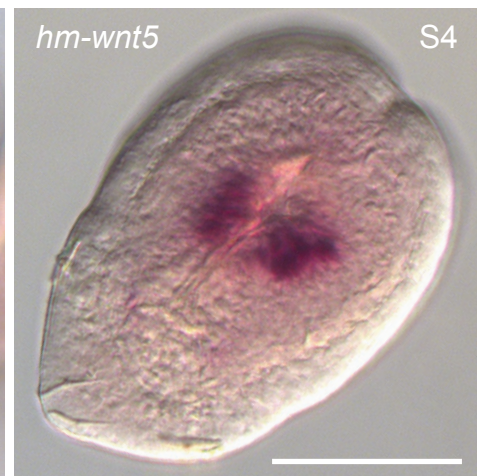

Supplement: Additional file 5: — Alkaline phosphatase-based development of whole-mount in situ hybridization in H. microstoma : Wnt2 and Wnt5. Bars: 50 μm. (PDF 1653 kb) [file 12915_2016_233_MOESM5_ESM.pdf]
